# Two's company: Monozygotic twinning in the small‐spotted catshark (*Scyliorhinus canicula*)

**DOI:** 10.1111/jfb.70049

**Published:** 2025-04-08

**Authors:** Andrej A. Gajić, Emilie de Loose, Andrea G. Martin, Elias Neuman, Emina Karalić, Hajrudin Beširović, Joel H. Gayford

**Affiliations:** ^1^ Sharklab ADRIA: Center for Marine and Freshwater Biology In‐Naxxar Malta; ^2^ Shark Measurements London UK; ^3^ IMBRSea Ghent University Ghent Belgium; ^4^ Department for Pathology, Faculty of Veterinary Medicine University in Sarajevo, (B‐H) Sarajevo; ^5^ College of Science and Engineering James Cook University Townsville Queensland Australia

**Keywords:** developmental abnormality, elasmobranch, fecundity, Mediterranean, oviparous, reproductive mode

## Abstract

Developmental abnormalities in elasmobranchs (sharks and rays) are rarely documented, with reported cases primarily involving fin or cranial deformities. Monozygotic twinning, the formation of identical twins from a single zygote, is particularly rare in these species and has been observed overwhelmingly in viviparous elasmobranchs. Here, we document a rare case of monozygotic twinning in an oviparous shark, the Small‐spotted Catshark *Scyliorhinus canicula* (Linneaus, 1758). This case was characterized by two embryos connected to a single yolk sac via separate yolk stalks within the egg capsule. The embryos exhibited normal development until mortality at developmental Stage 31 (Ballard et al., 1993) or stage 4 (Musa et al., 2018), likely due to stressors such as oxidative stress and allostatic overload, resulting from shared resources within the capsule. This is the first confirmed instance of mortality in monozygotic elasmobranch twins, highlighting the plausible challenges of polyembryony in oviparous elasmobranchs. These findings underscore the importance of understanding reproductive abnormalities and their implications for fecundity, particularly in light of ongoing anthropogenic pressures that threaten elasmobranch populations globally.

Although most vertebrate lineages are either oviparous or viviparous, elasmobranchs exhibit an array of reproductive strategies, including three varieties of oviparity, yolk‐sac viviparity, histotrophy, embryotrophy, oophagy and placental viviparity (Carrier et al., 2004; Katona et al., 2023; Musick et al., 2005), making them by far the most reproductively diverse vertebrate clade (Wourms, 1977). Despite the considerable variation in reproductive strategies among elasmobranchs, some commonalities unite these reproductive strategies, including internal fertilization, initial reliance on a yolk sac and meroblastic cell division during early development (Luer & Wyffels, 2022). Oviparity, the ancestral reproductive mode for all cartilaginous fishes (Katona et al., 2023; Mull et al., 2024), is exhibited by approximately 43% of extant elasmobranch species (Awruch, 2015; Hook et al., 2019). In oviparous species, egg cases are deposited in the external environment, typically containing a single embryo that depends entirely on its yolk sac for nourishment (Awruch, 2015; Carrier et al., 2004). The only known exceptions to this single‐embryo model occur in the big skate *Beringraja binoculata* (Girard, 1855) and the mottled skate *Beringraja pulchra* (Liu, 1932) (Ebert & Davis, 2007), where egg cases frequently contain three to four embryos. However, in this case each embryo possesses an independent yolk sac, a condition sometimes referred to as polyembryony (Chiquillo et al., 2014; Jang, 2019) or MEPE (“multiple embryos per eggcase”, see Gayford, 2025). This phenomenon highlights the variability and complexity of reproductive adaptations in oviparous elasmobranchs; however, monozygotic twinning, another rare developmental anomaly, remains undocumented in these species.

Although or polyembryony is a standard aspect of development and reproduction in *B. binoculata* and *B. pulchra*, twinning has occasionally been documented in other elasmobranchs, where it is typically considered a developmental abnormality (Luer & Wyffels, 2022). Most reported cases involve conjoined (bizygotic) twins with bicephaly (Sans‐Coma et al., 2017; Wakida‐Kusunoki et al., 2023), whereas some other morphological anomalies, such as duplicated mouths, eyes or misshapen appendages, have also been frequently described (Luer & Wyffels, 2022; Ramírez‐Amaro et al., 2019). In contrast, fewer than 15 cases of developmentally ‘normal’ twins have been recorded since 1885 (Ballard et al., 1993; Gudger, 1913; Hook et al., 2019; Ivanoff & Vooren, 2015; Lutz, 1976).

Historical records of twins in elasmobranchs are primarily associated with species using aplacental viviparity. These cases often reference distinct yolk sacs, suggesting bizygotic rather than monozygotic twinning (Gudger, 1913; Lutz, 1976). However, a more recent study by Ivanoff and Vooren (2015) documented suspected monozygotic twins in the shortnose spurdog *Squalus megalops* (Macleay, 1881), conjoined by a thin filament presumed to be of embryonic origin. Although the twin embryos were half the weight of their siblings, they exhibited a comparable rate of development (Ivanoff & Vooren, 2015).

Monozygotic twinning has also been reported in the oviparous clearnose skate (*Rostroraja eglanteria* Bosc, 1800). Observations made under controlled incubation conditions at Mote Marine Laboratory (Florida, USA) revealed that these twins, like those of *S. megalops*, were smaller than solitary embryos of the same developmental stage. Nevertheless, the twins successfully separated and exhibited normal behaviour following complete consumption of their shared yolk sac, suggesting that monozygotic twinning may not necessarily be a lethal developmental anomaly in elasmobranchs (Luer & Wyffels, 2022).

A similar case was presented by Lechenault et al. (1993) in the small‐spotted catshark *Scyliorhinus canicula* (Linnaeus, 1758), describing uniovular twins that were smaller in weight compared to solitary embryos. However, this report lacked photographic evidence or a detailed account of the development and eventual fate of the embryos. Additional instances of twinning in oviparous elasmobranchs have been reported (Hook et al., 2019), but none have provided conclusive evidence of monozygotic origin, highlighting the rarity and developmental complexity of such cases.

Given the extensive reproductive variation exhibited by elasmobranchs (Carrier et al., 2004; Conrath & Musick, 2012) and their precarious conservation status (Dulvy et al., 2021), understanding reproductive and developmental abnormalities – particularly those that impact fecundity – is critically important.

This study reports a novel rare case of monozygotic twinning in an oviparous shark, *S. canicula*, providing a detailed account of the developmental context and progression of the embryos under controlled conditions until mortality. The implications of this observation are examined, with a focus on the frequency of twinning in elasmobranchs, its physiological impacts on embryos and potential consequences for fecundity and conservation strategies.

On 7 February 2024, a total of 216 dead small‐spotted catsharks, including 31 adult females, were sampled as by‐catch from a commercial trawler operating out of Triport Harbour in Vlorë, Albania, at depths of 150–200 m. Each specimen was meticulously dissected following Crow and Brock (2004), with reproductive organs examined to determine the developmental stages of oocytes and identify any pathological conditions. Developed egg cases were carefully extracted from gravid females and incubated in controlled tanks, following Koehler et al. (2018), to facilitate proper embryonic development and hatching. Our research presented in this paper fully aligns with the Ethical Committee's recommendation, emphasizing the use of non‐protected, deceased specimens and adherence to the highest incubation standards for well‐being.

All egg cases were incubated in a 120‐L saltwater aquarium maintained at a constant temperature of 16°C, a salinity of 37.5‰ and an oxygen concentration of 8–10 mg/L. The system was equipped with continuous biological filtration and an oxygen pump to ensure optimal water quality. Egg cases and water parameters were monitored five times daily. On 11 March 2024, a single egg case was observed to contain two embryos, and their development was assessed using the embryonic staging tables provided by Ballard et al. (1993) and Musa et al. (2018). Although complete staging tables exist for only a few elasmobranch species, these references are appropriate for comparative purposes, as both studies utilize *Scyliorhinus* spp. as model taxa for oviparous elasmobranch embryonic development.

On 1 April 2024, the abnormal egg case was transferred from the original aquarium to a separate 50‐L aquarium maintained at a constant temperature of 17°C, a salinity of 37.5‰ and an oxygen concentration of 8–10 mg/L. Both embryos were photographed, and their morphological and behavioural characteristics were documented. After their death (details provided below), the embryos were carefully removed from the egg case using a small scalpel incision and preserved in 96% ethanol for subsequent analysis.

On 11 March 2024, dual embryos at stage 18–19 (Ballard et al., 1993) or Stage 3 (Musa et al., 2018) were identified in a single egg case, whereas all other egg cases contained single embryos at the same developmental stage. Both twin embryos were connected to a single yolk sac via separate yolk stalks, with no evidence of cleavage or separation between them (Figure 1). Each embryo displayed independent movement, capable of fully moving its body in a whip‐like manner. Their tails were elongated and pointed downwards, with clearly defined rostra. Thin red vitelline veins were visible on the shared yolk sac (Figure 1).

**FIGURE 1:**
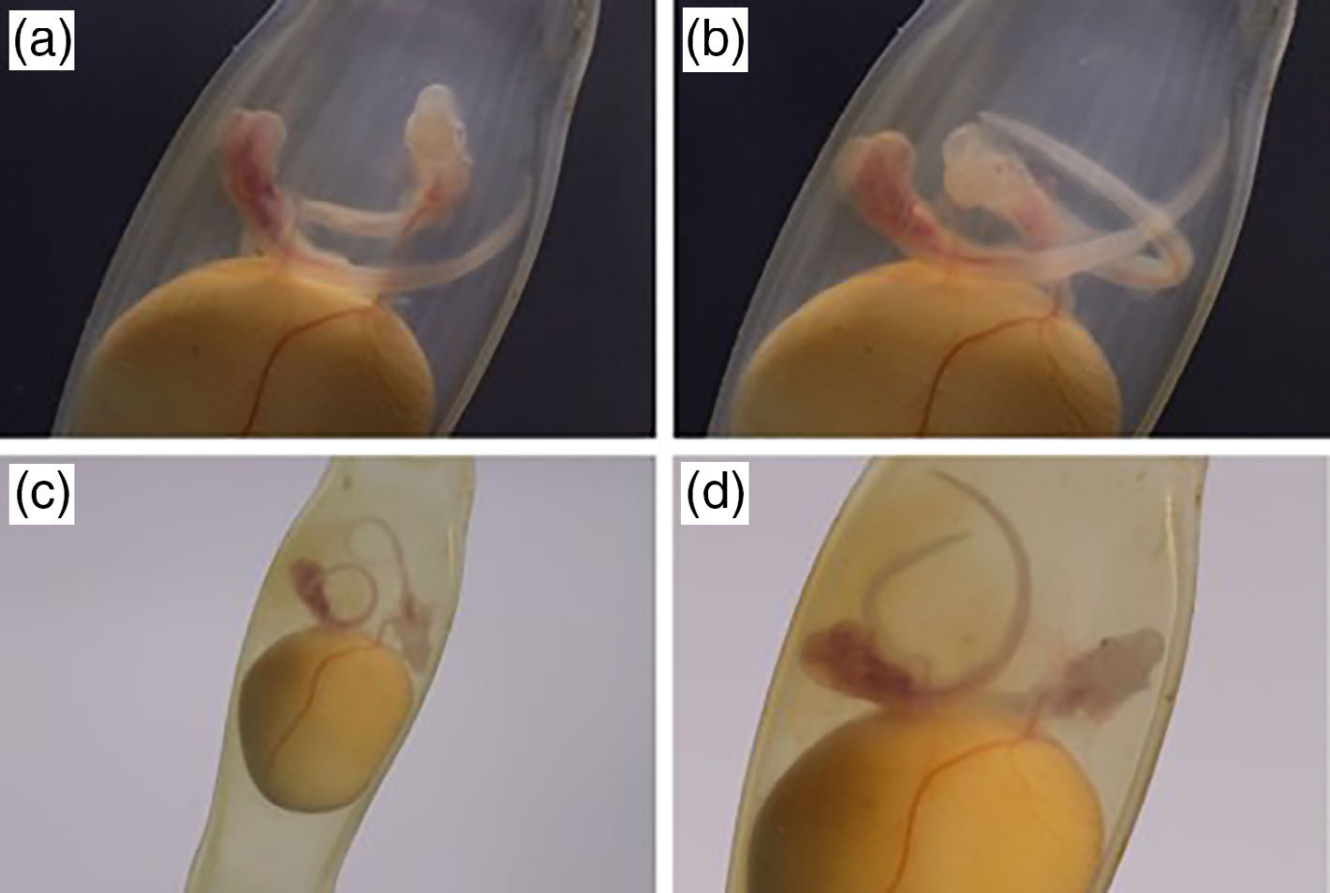
Monozygotic twins of *Scyliorhinus canicula* at Stage 30 (Ballard et al., 1993) or stage 4 (Musa et al., 2018), connected to a shared yolk sac. Notable differences include body colouration [red/pinkish in Twin 1 (left) vs. translucent in Twin 2 (right) (a–b) and more pronounced external gill filaments in Twin 1 (c–d)]. Photos: A. Gajić and M. Prelević.

By 28 March 2024, both embryos had progressed to stage 28, according to Ballard et al. (1993), or stage 4, according to Musa et al. (2018). Prominent external gill filaments were observed in all gill pouches of both embryos. The vitelline vein had developed into a striking red structure, with distinct veins supplying each embryo (Figure 1). Finfolds of the anal and pectoral fins had begun to form, and each branchial arch supported visible capillary veins. However, some notable morphological differences were observed between the embryos. Twin 1 exhibited more pronounced external gill filaments and a red‐pink body colouration, likely indicating stronger vascularization compared to Twin 2 (Figure 1). Conversely, the vitelline vein of Twin 1 appeared significantly smaller and thinner than the more prominent vein of Twin 2. Although the exact magnitude of vascularization differences could not be quantified, these variations might easily suggest differential physiological development between the twins.

On 1 April 2024, the embryos were observed to have reached Stage 30, according to Ballard et al. (1993), or stage 4, according to Musa et al. (2018). Key developmental features included dark pigmentation encircling the eyeballs, the curved final shape of the mouth, acute angles of the dorsal and anal fins and the protrusion of the rostrum anterior to the mouth (Figure 1). Twin 1 exhibited slower movements compared to Twin 2, maintaining a red‐pinkish colouration, whereas Twin 2 appeared more translucent and whiter. The disparity in the vitelline veins remained evident, with Twin 1's vein noticeably thinner.

On 3 April 2024, Twin 1 ceased all movement, collapsing onto the yolk sac with no observable ventilation attempts. After 5 h in this state, it was considered deceased, whereas Twin 2 remained active and showed no immediate signs of distress. However, on 4 April 2024, Twin 2 was also found deceased, exhibiting a similar condition to Twin 1. By this time, the body of Twin 1 had decomposed and was no longer distinguishable.

This study provides a detailed description of the development and mortality of *S. canicula* twins, contributing to the limited knowledge of monozygotic twinning in oviparous elasmobranchs. The presence of two embryos within a single egg case, joined to a shared yolk sac but exhibiting independent movements, strongly suggests a case of monozygotic twinning (Fenton et al., 2019; Ivanoff & Vooren, 2015; Luer & Wyffels, 2022). Developmental staging, according to Ballard et al. (1993) and Musa et al. (2018), indicated no deviations from typical growth patterns for *S. canicula*. Furthermore, both embryos were of similar size to each other and to solitary embryos in the same incubation conditions, aligning with known developmental benchmarks (Ballard et al., 1993; Musa et al., 2018).

The mortality of both embryos at approximately stage 30–31 (Ballard et al., 1993) raises significant questions about the physiological constraints associated with monozygotic twinning. This developmental stage corresponds with a period of hypoxia within the enclosed egg case, during which oxygen levels drop until mucous plugs dissolve, allowing water flow to restore normoxia (Ballard et al., 1993; Lechenault et al., 1993; Rodda & Seymour, 2008). In twin embryos, the combined oxygen demand likely exacerbated these hypoxic conditions, leading to heightened oxidative stress and allostatic overload (accumulation of lactate and carbon dioxide and acidosis due to pH alteration). This aligns with the hypothesis that gas exchange is limited by the shared yolk sac, which plays a dual role as a nutrient source and a respiratory structure (Rodda & Seymour, 2008; Rombough, 1989).

Importantly, although it was initially hypothesized that insufficient nutrient supply from the shared yolk sac might contribute to mortality, the presence of significant yolk reserves at the time of death suggests that other factors, such as inadequate gas exchange or excessive waste accumulation, were more critical. The smaller and thinner vitelline vein observed in Twin 1, alongside differences in vascularization, may have further compounded these challenges by reducing gas exchange efficiency and resource allocation compared to Twin 2 (Honda et al., 2022; Pelster, 2020; Rodda & Seymour, 2008). Additionally, the decomposition of Twin 1 likely elevated levels of nitrogenous compounds and other toxins within the egg case, creating a hostile environment that contributed to the death of Twin 2 (Mohan & Aiken, 2004; Ruiz‐Jarabo et al., 2022). This sequence of events underscores the physiological challenges of twin development within the constraints of an egg case evolved to support a single embryo.

Previous reports of monozygotic twinning in elasmobranchs, such as *S. megalops* (Ivanoff & Vooren, 2015) and *R. eglanteria* (Luer & Wyffels, 2022), described twins that were smaller than typical embryos but otherwise developmentally normal. In contrast, the twins in this study were of comparable size to solitary embryos but failed to survive beyond Stage 31. These differences highlight the potential influence of species‐specific factors, including egg case dimensions, yolk size and vascular architecture, on the viability of monozygotic twinning (Ebert et al., 2021; Porcu et al., 2017).

The findings of this study suggest that although a single yolk sac may provide sufficient nutrients for multiple embryos, the physiological demands of twinning – particularly for oxygen exchange – may exceed the capacity of the egg case in *S. canicula*. This contrasts with species such as *R. eglanteria*, where twins successfully absorbed their yolk sac and exhibited normal behaviour, suggesting that the viability of monozygotic twinning may vary significantly across elasmobranch species.

The rarity of monozygotic twinning in elasmobranchs underscores the need for further research into its frequency, viability and adaptive significance. Future studies should incorporate genetic assays to confirm monozygosity and investigate the developmental and physiological mechanisms underpinning twin development. Additionally, comparative studies across a broader range of elasmobranch species are critical to understanding the ecological and evolutionary implications of twinning.

In conclusion, this study advances our understanding of embryonic development in elasmobranchs and highlights the complex interplay between nutrient provisioning, oxygen exchange and physiological constraints in monozygotic twins. These findings serve as a foundation for future research into the mechanisms and ecological implications of polyembryony in elasmobranchs, with potential applications for conservation and management strategies.

## AUTHOR CONTRIBUTIONS

**Andrej A. Gajić:** review and editing, methodology, funding acquisition and supervision; **Emilie de Loose:** writing – original draft; **Andrea G. Martin:** data collection and data analysis; **Elias Neuman:** data collection and data analysis; **Emina Karalić:** data collection and data analysis; **Hajrudin Beširović:** data analysis and review and editing; **Joel H. Gayford:** conceptualisation and writing – original draft. All authors contributed to editing and review of the final manuscript.

## FUNDING INFORMATION

This work was supported by the 5th Completion grant funded by Rufford Foundation, awarded to Andrej A. Gajić, for the project ‘Effective Conservation and Research of Threatened Sharks, Skates and Rays through Rescue, Rehabilitation, Tagging and Re‐Introduction’.
